# Magnesium Slag-Activated One-Part Geopolymer Concretes: A Viable Supplementary Pathway Toward Low-Carbon Concrete Production

**DOI:** 10.3390/ma19030551

**Published:** 2026-01-30

**Authors:** Tuğba Özdemir Mazlum, Nihat Atmaca

**Affiliations:** 1 Graduate School of Natural and Applied Sciences, Gaziantep University, Gaziantep 27310, Türkiye; 2 Civil Engineering Department, Gaziantep University, Gaziantep 27310, Türkiye; atmaca@gantep.edu.tr

**Keywords:** alkali-activated binders, industrial by-products, solid activators, sustainability, compressive strength, microstructure

## Abstract

Amid growing environmental concerns, resource depletion, and the pressing challenges of industrial waste management, this study investigates the potential of magnesium slag (MS) as a sustainable alternative binder in the production of one-part geopolymer concretes (OPGCs). The objective is to reduce reliance on conventional cementitious materials while promoting the valorization of industrial by-products in construction practices. For this purpose, ten different mixtures were designed by replacing ground granulated blast furnace slag (GGBS), the conventional aluminosilicate precursor, with MS, an innovative aluminosilicate precursor, at replacement levels of 10%, 20%, 30%, 40%, 50%, 60%, 70%, 80%, 90%, and 100% by weight, using a solid activator. The fresh and hardened properties of these mixtures were systematically evaluated through slump, setting time, density, ultrasonic pulse velocity (UPV), and strength tests, while microstructural characterization was also conducted using scanning electron microscopy (SEM) coupled with energy-dispersive X-ray spectroscopy (EDX) to further investigate the geopolymerization process, elemental distribution, and the role of MS in binder formation in OPGC. The results revealed that MS incorporation significantly influenced both workability and mechanical performance, and it was confirmed that MS actively participates in geopolymerization and can be effectively utilized up to a certain threshold. Replacement levels up to 30% were found to maintain acceptable mechanical performance, providing evidence that MS is a promising precursor for developing sustainable OPGC.

## 1. Introduction

The production process of Portland cement-based concrete requires a significant amount of natural resources and substantially contributes to greenhouse gas emissions [1]. Scientists and researchers continuously explore the potential use of waste materials that can offer similar or even superior performance compared to cement-based concrete [2]. This approach aims to promote waste recycling, reduce CO_2_ emissions, and conserve natural resources [3]. Over the past two decades, there has been growing interest in developing environmentally friendly construction materials by incorporating industrial by-products, such as supplementary cementitious materials or fillers. The superior performance of these materials is attributed to the morphological and pozzolanic properties of supplementary cementitious materials. These properties also play a crucial role in enhancing the structure of the cement-based concrete matrix [4].

Geopolymer concretes have emerged as a significant innovation capable of replacing ordinary Portland cement-based concrete. By using the correct geopolymer mixture, CO_2_ emissions can be reduced by up to 80% and energy consumption by up to 60% compared to conventional cement [5]. As a result, geopolymers have gained considerable interest and recognition among researchers. The term “geopolymer” was first defined by Davidovits in 1979 [6]. Traditional geopolymer formulations typically consist of a two-component system, which includes a liquid phase (activator) and a solid phase (aluminosilicate materials). Geopolymer concretes offer numerous advantages, such as being environmentally friendly, providing high workability, and exhibiting high compressive strength [7]. They also demonstrate superior resistance to acids and sulphate [8], excellent thermal resistance [9], and low shrinkage during drying with minimal creep [10]. However, the geopolymerization process is complex and difficult to combine [11]. Additionally, there are some challenges associated with geopolymer concrete materials, such as the transportation, storage, and application difficulties of alkali solutions. These solutions are viscous, difficult to transport, and challenging to store in large quantities [12]. Since the soluble silicates used in geopolymer concrete are not entirely consumed during the geopolymerization process, this increases the permeability of conventional geopolymer concretes and reduces their durability. Therefore, the further development and improvement of two-part geopolymer concretes were necessary until the discovery of one-part geopolymer concretes.

The development of one-part geopolymer concretes (OPGC) has led to significant progress in the production of geopolymer concretes. These types of geopolymer concretes require only the addition of water during application. OPGC consist of solid activators and alumina–silica components. These components are thoroughly mixed before use, and water is added as needed, similar to Portland cement [13]. This innovation eliminates the need for the alkaline solutions traditionally used in two-part geopolymers. One-part geopolymer mixes offer significant practical advantages over traditional two-part systems, primarily through the simplification of the preparation process, which eliminates the need for handling large quantities of corrosive alkaline solutions on-site. Furthermore, this approach ensures a more uniform performance by reducing human errors during mixing, thereby enhancing the reliability and commercial viability of sustainable binders in large-scale architectural applications. Initially, some researchers identified issues such as low compressive strength [14] and poor mechanical properties [15] in one-part geopolymer concretes. However, extensive research was conducted to improve the geopolymer concretes. These studies led to increased compressive strengths of geopolymer concretes at optimal temperatures, making them competitive with traditional geopolymer concretes. Solid sodium metasilicate (Na_2_SiO_3_) and solid sodium hydroxide (NaOH) are highly effective powder activators in the formulation of one-part geopolymer mixtures [16]. In addition to that, some studies showed the anhydrous Na_2_SiO_3_ is a more effective solid activator in the metakaolin and fly ash mixture compared to the combination of hydrated Na_2_SiO_3_ and hydrated NaOH [17]. Also, some studies demonstrated that one-part geopolymer concrete containing a hybrid of solid Na_2_SiO_3_ and solid Na_2_CO_3_ exhibited the best overall performance in terms of mechanical performance, energy consumption, environmental impact, and economic potential [18]. A review of previous research also revealed that the performance of the innovative one-part geopolymer concrete is significantly influenced by the type of the aggregates used in the geopolymer mixtures [19].

Magnesium slag (MS) is a by-product generated during the production of magnesium or ferroalloys, particularly from thermal reduction processes involving dolomite or magnesite. Chemically, MS predominantly contains magnesium oxide (MgO), calcium oxide (CaO), silicon dioxide (SiO_2_), and aluminum oxide (Al_2_O_3_), along with smaller quantities of iron oxides and other trace elements [20]. The relatively high contents of reactive silica and alumina in MS provide potential for its use as an aluminosilicate precursor in geopolymer synthesis [21]. In addition, the presence of free lime (CaO) and periclase (MgO) may contribute to secondary hydration or carbonation reactions, which can influence setting time, dimensional stability, and mechanical performance [22]. Although MS is less reactive compared to conventional precursors like GGBS or fly ash (FA), previous studies have shown that, under alkaline activation, MS can partially participate in geopolymerization reactions and improve sustainability by reducing reliance on primary resources and mitigating waste disposal issues [23]. Some studies have made significant attempts to conduct more comprehensive research on the CO_2_ curing of MS. These investigations aim to understand the influence of carbonation parameters—such as CO_2_ concentration, curing duration, and temperature—on the microstructural development, strength evolution, and carbon fixation capacity of MS-based binders. Recent findings suggest that CO_2_ curing not only enhances early strength, but also promotes the formation of stable carbonate phases, contributing to both durability improvement and environmental sustainability [24]. However, there is a lack of research focusing on the incorporation of MS in one-part geopolymer concretes. This study seeks to fill this gap in the literature by systematically examining the feasibility and performance of MS in one-part geopolymer concrete mixes.

Another type of the alumina–silicate materials used in geopolymer concrete production, GGBS, is a by-product formed during the processing of iron in the blast furnace and is an important component for geopolymer concrete due to its chemical and physical properties [25]. With its high content of reactive silica and alumina, it makes the concrete suitable for forming strong chemical bonds with alkaline activators during the geopolymerization process [26]. This process contributes to the formation of a dense and durable matrix, enabling geopolymer concrete to exhibit superior resistance to sulphate attacks, acid corrosion, and alkali–silica reactions [27]. These properties make geopolymer concrete containing GGBS an ideal material for structures exposed to harsh environmental conditions. Moreover, the low permeability of GGBS minimizes the penetration of water and harmful ions such as chlorides into the concrete matrix, thereby enhancing the long-term durability of structures [28]. The reduction in the permeability of geopolymer concrete not only helps reduce environmental impacts, but also lowers maintenance and repair costs [29]. In terms of mechanical performance, GGBS significantly increases the compressive strength of geopolymer concrete both in the early stages and over the long term [30]. GGBS plays a reactive role in the geopolymerization process due to its high content of calcium silicate, alumina, and other active components, contributing to the improvement in durability and mechanical properties in the concrete [31]. With its high hydraulic potential, GGBS reacts with alkaline activators to form a binder phase that strengthens the microstructure of the geopolymer matrix [32]. The use of GGBS in geopolymer concrete significantly reduces permeability, limiting the ingress of harmful ions and providing additional protection against corrosion [33]. Environmentally, the use of GGBS supports sustainability goals by reducing reliance on natural raw materials [34]. The use of GGBS as a binder in geopolymer concrete production significantly reduces the need for Portland cement, thus contributing to the reduction of carbon emissions from cement production. Additionally, utilizing industrial wastes such as MS and GGBS as a building material help mitigate waste management issues and contribute to the circular economy. Extensive research has been carried out on industrial wastes such as granulated blast furnace slag, fly ash, and steel slag, offering valuable insights into their potential reuse in traditional geopolymer concrete. Yet, studies addressing the cementitious performance of magnesium slag as a precursor in one-part geopolymer concrete are relatively limited, which contributes to its underutilization. This highlights the urgent necessity of investigating its structural properties and identifying pathways for more efficient application.

The primary objective of this research is to investigate the feasibility of utilizing MS as a sustainable precursor aluminosilicate source in one-part geopolymer concrete systems, thereby addressing the dual challenges of industrial waste valorization and the reduction in the carbon footprint associated with traditional cement production. By investigating the synergistic effects of MS and GGBS blends, this study seeks to establish a fundamental understanding of how MS incorporation influences the geopolymerization process and the resulting structural integrity. To achieve this, the study systematically analyzes a range of substitution levels to identify optimal mix designs that balance environmental benefits with engineering properties.

To clarify the framework of this research, the boundaries of the study are defined by the systematic replacement of GGBS with MS at ten incremental levels (from 10% to 100%) within a one-part geopolymer system using a solid sodium metasilicate activator. The primary hypothesis of this study is that MS, despite its lower reactivity compared to GGBS, can actively participate in the geopolymerization process and serve as a viable aluminosilicate precursor up to a specific replacement threshold without compromising the structural integrity of the concrete. Based on this hypothesis, the study addresses the following research questions:

- To what extent can MS replace GGBS in OPGC while maintaining acceptable mechanical performance and workability?
- How does the incorporation of MS influence the setting time and the evolution of the microstructural network (C–A–S–H/N–A–S–H gels)?
- Is there a critical threshold where the reduction in reactive silica and alumina from MS significantly disrupts the matrix cohesion?

The remainder of this article is organized as follows: Section 2 details the materials used and the experimental methodology, including the design of the ten distinct OPGC mixtures. Section 3 presents and discusses the experimental results regarding fresh state properties, mechanical strength development, and durability performance. Finally, Section 4 summarizes the key findings and provides concluding remarks on the potential of MS-based geopolymers for low-carbon construction.

## 2. Experimental Program

The experimental program of this study is strategically designed to evaluate the maximum feasible incorporation of MS within a one-part geopolymer framework. The selection of raw materials and the formulation of the experimental matrix have been rigorously planned to establish a robust scientific basis for the mixtures. By conducting a comprehensive characterization of both fresh and hardened state properties, this methodology aims to provide a fundamental understanding of the material’s structural behavior and overall performance.

### 2.1. Materials and Mix Design

The materials used in this research were selected based on their chemical composition and reactivity to ensure an effective geopolymerization process. MS was chosen as the primary innovative precursor due to its high MgO content and potential for waste valorization, while GGBS was integrated to provide the necessary aluminosilicate base for early-strength development.

**Figure 1:**
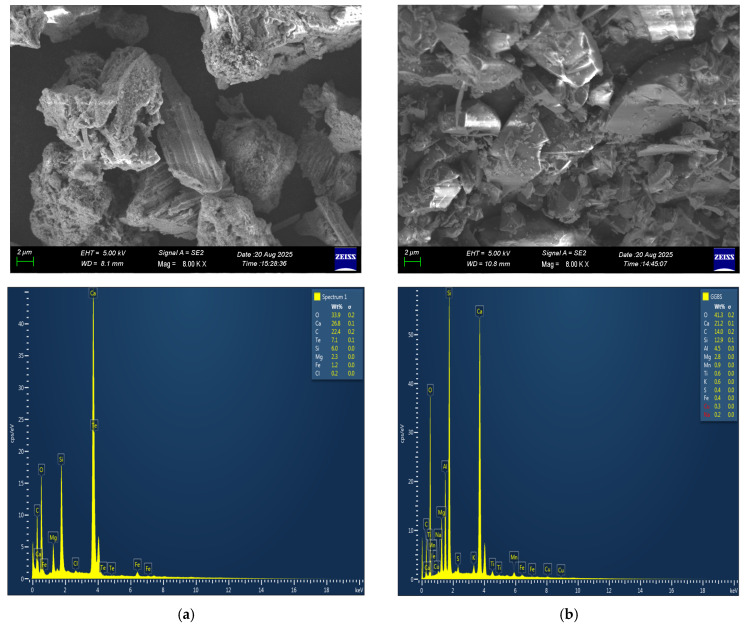
SEM-EDX images of (**a**) MS; (**b**) GGBS.

MS, serving as the aluminosilicate precursor for one-part geopolymer concrete, was supplied by Kar Mineral Mining Inc., Eskişehir, Turkey. The GGBS used in the experiment was provided by the Çimko Cement and Concrete Factories Inc., Gaziantep, Turkey. The chemical compositions provided by the XRF (X-ray fluorescence) analysis of these materials are presented in Table 1. The chemical analysis indicated that the amount of alumina (Al_2_O_3_) in MS was lower than in GGBS, while the silica (SiO_2_) contents were found to be nearly identical, recorded as 29.68% and 29.40%, respectively. In addition, the calcium (CaO) concentration in MS was reported to be higher than that in GGBS. The unit weights of the MS and GGBS used in the experiment were 2.16 and 2.86 g/cm^3^, respectively. SEM microstructure images of the MS and GGBS are also shown in Figure 1. The micrographs reveal notable surface irregularities and a lack of compact, dense structures, indicating heterogeneous and porous morphological characteristics.

The solid activator sodium metasilicate (Na_2_SiO_3_) was obtained from Songen Biotechnology and Laboratory Materials Ltd. Co., Istanbul, Turkey, and its physical and chemical properties are shown in Table 2.

**Figure 2:**
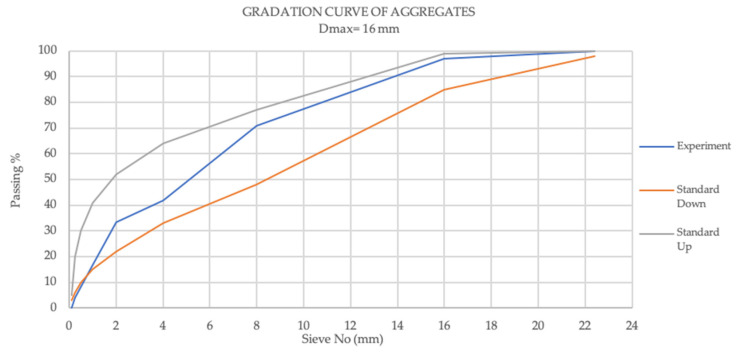
The gradation curve of the aggregates used in the experiment.

The aggregates, consisting of both fine and coarse aggregates, were sourced from KÇS Cement, Gaziantep, Turkey. The particle sizes of the aggregates incorporated into the concrete mixtures are within the boundaries outlined according to the EN 12620 standard in Figure 2 [35]. The first category includes coarse aggregates with a maximum particle size (Dmax) of 16 mm, while the second category consists of fine aggregates with a fineness modulus of 4.37. The water absorption rates, the dry and saturated surface, and dry-specific gravities of the aggregates used in the experiments are presented in Table 3.

OPGC was produced using water, coarse-fine aggregates, and alternative cement (raw material and activator). The primary raw materials used were MS and GGBS, while solid Na_2_SiO_3_ was employed as the activator. Considering the similarities between conventional concrete and OPGC, the mixture design method was adapted based on previous studies [19]. The quantities used in the OPGC experiment are presented in Table 4. According to this table, the alumina–silicate materials represent MS and GGBS, while the binder consists of alumina–silicate materials and a solid activator.

In this experimental OPGC mixture, the total aggregate content of the concrete was set at 70% by mass, with coarse and fine aggregates constituting 60% and 40% of the total aggregate, respectively. The ratio of solid activator to alumina–silicate materials was kept at 0.16 and the ratio of water to binder materials was kept constant as 0.47. This experimental study aims to investigate the feasibility of using MS in OPGC. Therefore, the experimental tests of each specimen were analyzed by adding MS to the GGBS based OPGC in specific proportions. In this context, MS1 represents the specimen containing 10% MS and 90% GGBS with the remaining proportions determined in a similar manner, thereby forming the table presented in Table 5.

### 2.2. Preparation of Specimens and Curing Conditions

**Figure 3:**
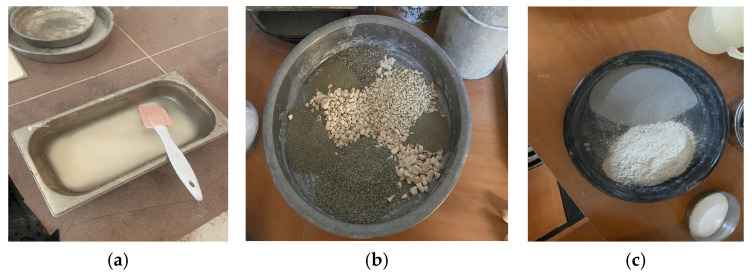
Mixing and curing stages of MS-based OPGC: (**a**) dissolving of solid activator in the water, (**b**) coarse and fine aggregates, (**c**) MS (up) and GGBS (bottom) used for experiment.

A total of 270 specimens, comprising 27 from each of ten different mixes, were produced to assess the feasibility of incorporating MS into OPGC. Among these, three specimens were designated for 7-day testing and another three for 28-day testing. Each set of 7-day and 28-day specimens included one cube, one cylinder, and one prism type, with three identical specimens cast for each to allow for averaging the results. The mixing process was carried out a laboratory-scale pan mixer with a 75 L capacity and a rotational speed of 280 rpm. Initially, the coarse and fine aggregates were dry-mixed for approximately 2 min. Subsequently, the MS and GGBS was added to the mixture, and mixing continued for another 2 min to ensure uniform distribution of the dry materials. Once a consistent dry mix was achieved, the solid activator was introduced by dissolving in the mixing water to enhance the homogeneity of the mixture and ensure the better penetration of the activator into the other solid constituents, as shown in Figure 3. The mixture was then blended for an additional 6 min until a uniform consistency was obtained. Slump tests were carried out directly after mixing, in accordance with EN 12350-2, to evaluate the workability of the fresh concrete mixtures [36]. The test was conducted under ambient temperature conditions of 20 ± 2 °C. The final fresh mixture, which followed a process similar to conventional concrete production, and thus offers practical advantages in terms of ease of handling, was cast into molds (oiled before use) for experimental testing. Cube specimens (150 × 150 × 150 mm), cylindrical specimens (100 × 200 mm), and prism specimens (100 × 100 × 400 mm) were prepared accordingly. After casting into the molds, the specimens were compacted with the aid of a vibrator to ensure proper consolidation and eliminate entrapped air. After casting and vibrating, the specimens were covered with heat-resistant plastic wrap to prevent moisture loss and then placed in a furnace set at 60 °C for thermal curing. This temperature was selected to ensure optimal geopolymerization while preventing internal micro-cracking caused by rapid moisture loss at higher temperatures [37]. Moreover, the purpose of this process was to accelerate the chemical reactions among the constituents of the geopolymer concrete, thereby ensuring timely setting. As part of the preliminary study, specimens without thermal curing were also prepared. It was observed that these specimens exhibited delayed setting, and those demolded prematurely lost their structural integrity. Therefore, thermal curing was deemed essential for the proper setting of one-part geopolymer concrete. The specimens remained in the furnace for 24 h. Upon completion of thermal curing, they were removed from the furnace, the plastic wrap was taken off, and the specimens were sealed in plastic bags to retain their internal moisture. They were then stored at ambient temperature until the designated testing days.

### 2.3. Testing Method

A comprehensive testing program was established to characterize the performance of MS-based OPGC in terms of various properties. Fresh state tests were conducted to evaluate workability and practical application limits, while mechanical and durability assessments were specifically integrated to evaluate the influence of MS on the compressive strength development and the structural stability of the geopolymer matrix.

**Figure 4:**
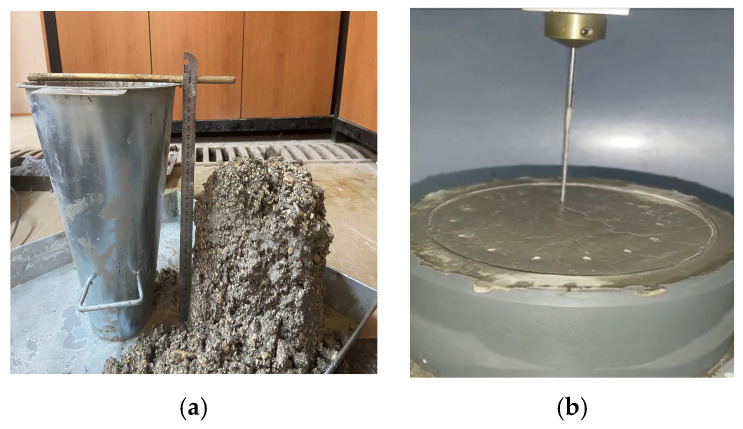
Experimental test procedures of fresh MS-based OPGC: (**a**) slump test; (**b**) determination of setting time.

The slump test was performed to assess the workability of fresh OPGC mixtures, as shown in Figure 4a. The test followed EN 12350-2 standard procedures and was conducted immediately after mixing. The primary objective was to evaluate the consistency and flow behavior of the mixtures containing varying proportions of MS and GGBS. Due to the absence of Portland cement and the unique setting characteristics of geopolymer systems, their fresh-state behavior can differ significantly from conventional concrete. Therefore, the slump test served as an essential indicator to determine whether the mixtures had adequate flowability for proper casting and compaction. The results provided valuable insight for optimizing mix proportions in relation to fresh concrete performance.

The setting time of MS-based OPGC were determined in accordance with ASTM C807-21 standard, as shown in Figure 4b [38]. For this purpose, mortar was obtained by sieving fresh concrete through a #4 mesh to remove coarse aggregates. The setting time was recorded from the moment the aluminosilicate materials came into contact with the activator until penetration of a 2 mm diameter needle was less than 10 mm. The reported values for setting time were derived from the average of two specimens.

**Figure 5:**
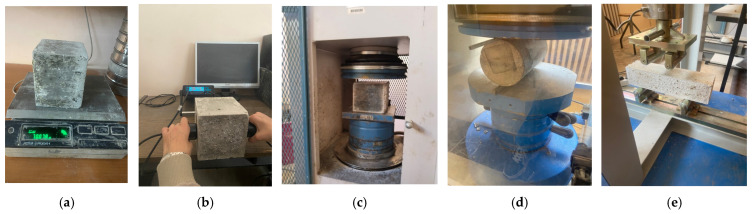
Experimental test procedures of hardened MS-based OPGC: (**a**) measurement of specimen weight for density determination; (**b**) ultrasonic pulse velocity (UPV) test; (**c**) compressive strength test on cube specimen; (**d**) splitting tensile strength test on cylindrical specimen; (**e**) flexural strength test on prismatic specimen.

The density of hardened OPGC was determined to evaluate the compactness and uniformity of the specimens in accordance with EN 12390-7 standard [39]. After 28 days of curing, cubic samples were dried to a constant weight and their mass and volume were recorded as seen in Figure 5a. The density was calculated by dividing the mass of the specimen by its volume, following standard procedures. This simple but important measurement provides insight into the internal structure of the concrete, indicating the degree of compaction and potential presence of voids. A consistent and relatively high density value suggests effective particle packing and good quality in the hardened state.

The ultrasonic pulse velocity (UPV) test was carried out on 28-day-cured OPGC specimens to assess their internal quality in a non-destructive manner. The test was conducted in accordance with the EN 12504-4 standard using a portable UPV device equipped with two transducers [40]. A coupling gel was applied to the surface of the specimens to ensure proper contact and signal transmission. The transducers were positioned on opposite faces of the concrete sample as seen in Figure 5b, and the time taken for the ultrasonic pulse to travel through the material was recorded. The pulse velocity was calculated by dividing the specimen length by the travel time. Higher pulse velocities generally indicate a denser and more homogeneous structure, while lower values may suggest internal flaws, voids, or microcracks.

The compressive strength of the concrete cube specimens (150 × 150 × 150 mm) was determined in accordance with the EN 12390-3 standard [41]. Prior to testing, the surface of each specimen was cleaned and aligned carefully to ensure a uniform load distribution during testing. A calibrated compression testing machine, Utest, in Mechanical Laboratory of Gaziantep University, Gaziantep, Turkey, was used with a capacity of 2000 kN to apply the load at a constant pace rate of approximately 0.6 MPa/s until failure, as shown in Figure 5c. The average value of three specimens was recorded as the compressive strength for each mix design at early age strength as 7 days, at standard age strength as 28 days, and at long-term strength as 90 days.

The splitting tensile strength of concrete specimens was determined in accordance with the EN 12390-6 standard [42]. The cylindrical specimens, with dimensions of 100 × 200 mm, were placed horizontally between the loading platens of the testing machine, as shown in Figure 5d. A compressive load was applied along the vertical diameter of each specimen using strips to ensure uniform stress distribution. The load was applied at a constant rate of 0.05 MPa/s until failure occurred. The maximum load at failure was recorded, and the splitting tensile strength was calculated accordingly. The tests were performed at 28 days of curing, and the average value of three specimens was reported for each age.

The flexural strength of prism specimens (100 × 100 × 400 mm) was assessed following the third-point loading method specified in EN 12390-5 [43]. Specimens were positioned on two supporting rollers spaced 30 cm apart, and the load was applied through two loading rollers placed symmetrically at one-third span points, as shown in Figure 5e. The test was conducted using a universal testing machine, Utest, in Mechanical Laboratory of Gaziantep University, Gaziantep, Turkey, at a loading rate of 0.1 MPa/s until failure occurred with 28 days of specimen curing. The flexural strength was calculated based on the maximum load and span dimensions, and the average of three specimens was taken as the representative value.

In the final stage, SEM (scanning electron microscopy) and EDX (energy-dispersive X-ray spectroscopy) analyses were carried out to examine the microstructural and chemical characteristics of the specimens. The SEM analysis provided high-resolution images of the surface morphology and pore structure, offering detailed insights into the distribution of hydration products, microcracks, and bonding zones. In this study, the SEM device used was the Zeiss Gemini SEM 300, Gaziantep University Uluğ Bey High Technology Application and Research Center (ULUTEM), Gaziantep, Turkey. Furthermore, with the aid of the EDX detector integrated into the SEM system, the elemental composition of the material was identified; in particular, the distribution and proportions of elements such as Si, Al, Ca, Mg, and Fe—which play a critical role in the geopolymerization process—were determined. Thus, a comprehensive assessment was achieved from both morphological and chemical perspectives, clearly revealing the influence of the raw materials (MS and GGBS) on the formation of reaction products.

## 3. Results and Discussion

This section presents the experimental findings alongside a comprehensive discussion to evaluate the influence of MS on the properties of OPGC. By integrating results with discussion, the study aims to establish a direct correlation between the observed physical behaviors and the underlying geopolymerization mechanisms, providing a more cohesive understanding of the material’s performance.

### 3.1. Workability

Table 6 shows the workability results for the MS-based OPGC. All mixtures exhibited a satisfactory level of workability. In general, the mixtures exhibited similar slump values, but workability showed an upward trend increasing the proportion of MS. This situation is explained by the mineralogical composition of magnesium slag. The silicon present in MS is largely in the form of Q^0^ and Q^4^ units, which are associated with a high number of bridging oxygens and a strong degree of [SiO_4_] polymerization. This structural characteristic results in relatively lower inherent cementitious activity compared to the GGBS, where silicon is predominantly present in Q^0^ units with fewer bridging oxygens and a lower polymerization degree, providing higher reactivity. However, when exposed to alkali activation, the latent cementitious potential of MS becomes more evident, although it is still significantly lower than that of GGBS. This microstructural behavior explains why mixtures with higher MS content show improved workability despite their lower cementitious reactivity.

### 3.2. Setting Time

**Figure 6:**
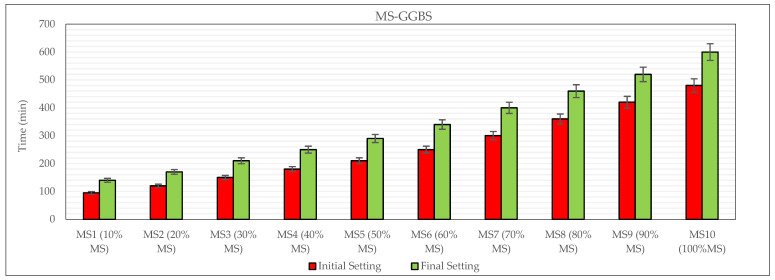
Initial and final setting times of MS-based OPGC.

The initial and final setting times results of MS-based GGBS are shown in Figure 6. The initial and final setting times increased progressively with the rise in MS content. In the MS1–MS3 range, the initial setting occurred within approximately 100–150 min, indicating a relatively rapid hardening behavior. From MS6 onwards, a marked increase in setting times was observed, with the final setting of MS10 reaching 600 min. It has been shown that MS markedly extends the setting time in geopolymer systems. This behavior is attributed to the lower reactivity of MS and the enhanced reaction kinetics provided by GGBS. According to this graph in Figure 6, when the GGBS was 90% replaced by MS, the duration of the initial and final set was extended from 95 to 480 min (405%) and from 140 to 600 min (320%), respectively. This aligns with earlier studies indicating that increasing the proportion of GGBS in the binder leads to a reduction in setting time [44,45,46,47,48]. It also agrees with the initial and final setting times reported in other studies [49].

Overall, the general tendency was that higher MS replacement resulted in longer setting times, whereas lower MS levels led to shorter setting times.

### 3.3. Wet Apparent Density

The wet apparent density of each mixture was measured at 28 days prior to conducting the UPV and strength tests. The results revealed that the density of OPGC mixtures decreased progressively with increasing MS content. Mixtures incorporating 10% MS measured the highest density (2.28 g/cm^3^), whereas those with 100% MS reached the lowest value (2.10 g/cm^3^). The observed decrease in density is attributed to the adverse effect of MS on the particle size distribution within the mixture, which agrees with the findings reported by a previous study [50]. This condition hindered the dense and efficient packing of particles, leading to an increase in the void sizes within the geopolymer concrete mixture. Compared to GGBS that is more reactive and denser binder, MS contributes to a reduced overall density. As a result, the mixture containing 10% MS was denser by about 7.9% compared to the mixture incorporating 100% MS.

**Figure 7:**
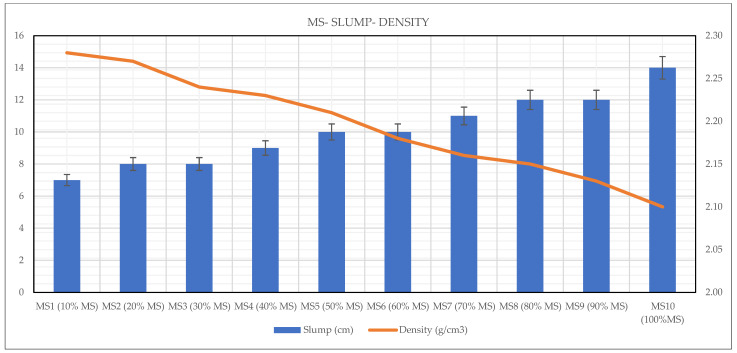
Wet apparent density vs. slump results of MS-based OPGC.

Moreover, the slump–density curve presented in Figure 7 revealed an inverse relationship between these two parameters. Mixtures with higher densities (≥2.27 g/cm^3^) exhibited lower slump values (7–8 cm), indicating stiffer and less workable mixes. On the other hand, as density decreased, slump values increased, with the most fluid mixture (100% MS) reaching a slump value of 14 cm. However, this increased flowability did not reflect improved quality; instead, it indicated the presence of excessive free water and entrapped air due to insufficient reactivity and lack of cohesion. These findings demonstrate that both excessively high and low MS contents can negatively affect the fresh performance of geopolymer concrete. Therefore, maintaining a balanced ratio of MS and GGBS is essential to achieve optimal density and workability, which are critical for the production of durable and structurally sound geopolymer concretes.

### 3.4. Ultrasonic Pulse Velocity (UPV)

**Figure 8:**
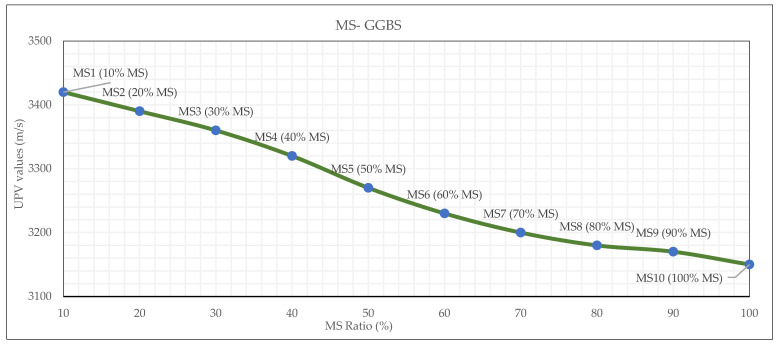
UPV test results of MS-based OPGC.

The UPV test of each mixture was measured at 28 days prior to conducting strength tests. The UPV test results show a clear decrease as the MS ratio increases in the OPGC mixtures. As shown in Figure 8, UPV values dropped from 3420 m/s at 10% MS to 3150 m/s at 100% MS, reflecting a gradual loss in ultrasonic wave transmission capacity through the material matrix. This decrease in UPV is associated with the higher porosity and reduced density of MS when incorporated at increased proportions. Compared to more reactive and denser materials like GGBS, MS tends to form less cohesive and more voided structures, which disrupt the continuity of the solid matrix and slow down wave propagation. In geopolymer systems, high UPV values generally correlate with good microstructural integrity, low porosity, and strong inter-particle bonding. Therefore, the observed decline in UPV suggests that increasing the MS content leads to a weaker internal structure, increased microvoids, and reduced bonding quality.

### 3.5. Compressive Strength

**Figure 9:**
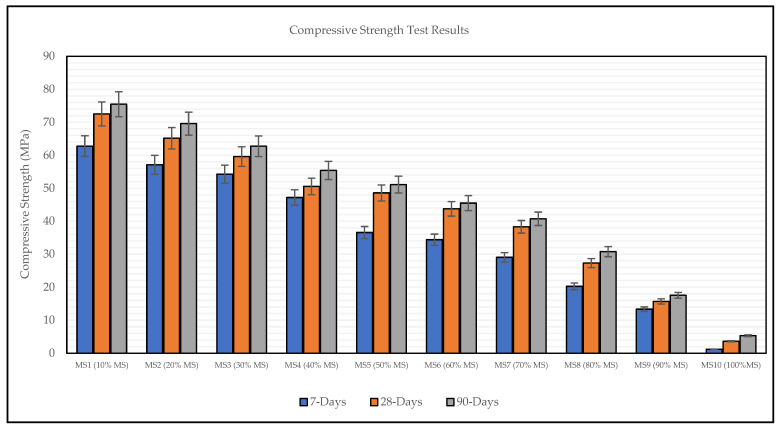
Compressive strength test results of MS-based OPGC.

The compressive strength test results of MS-based OPGC mixtures are illustrated in Figure 9. Each value presented represents the average of at least three tested specimens. The obtained results indicate that the proportion of MS in the OPGC mixtures played a crucial role in the development of compressive strength. The 7-day strength of the samples ranged from 1.20 to 62.78 MPa, the 28-day strength varied between 3.60 and 72.53 MPa, and the 90-day strength ranged from 5.30 to 75.48 MPa, as shown in Figure 8. The highest compressive strength was recorded for the mixture incorporating 10% MS, which exhibited strength characteristics comparable to those of high-performance conventional concretes. As shown in following Figure, increasing the MS content from 10% to 100% progressively reduced the compressive strength at all curing ages (7, 28, and 90 days).

The geopolymerization process begins with the decomposition of solid aluminosilicate materials into silicate and aluminate monomers, which subsequently undergo condensation reactions to form the geopolymer gel. Moreover, an increased calcium content in geopolymer concrete has been reported to facilitate the dissolution of aluminosilicates by elevating the pH of the surrounding medium, thereby enhancing the degree of geopolymerization and promoting the development of a stronger geopolymer network [51]. The rise in compressive strength resulting from the reduction in MS content can be linked to the balanced effect of soluble silica originating from both the activator and GGBS. This interaction enhances the strength development at different curing ages. In contrast, mixes composed solely of MS (100% MS) demonstrated restricted strength gain, as verified by these experimental findings. Additionally, the gradual improvement in strength could be related to the chemical reaction between aluminosilicate phases and the solid activator, which facilitates the dissolution of silica and alumina and accelerates the geopolymerization mechanism [52]. When the MS content reached 30%, the overall silica and alumina concentration in the binder matrix decreased rapidly due to the nature of MS. Consequently, the number of Si–O–Al bonds, especially between MS and GGBS particles, was reduced, leading to a drop in compressive strength. Moreover, the relatively low strength of the mixtures containing 100% MS can be attributed to the insufficient calcium content, which limits the dissolution and subsequent gel formation [53].

**Figure 10:**
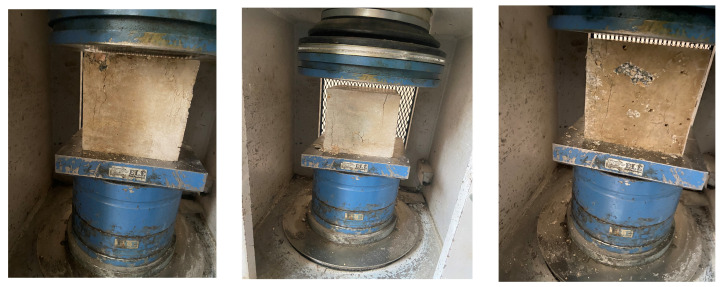
Fractured specimens of hardened concrete after the compressive strength test.

It is noteworthy that the obtained results are consistent with those of previous studies [54,55], in which researchers investigated the influence of MS in traditional concrete. Moreover, Figure 10 shows the fractured specimens of hardened concrete after the compressive strength test.

### 3.6. Splitting Tensile Strength

**Figure 11:**
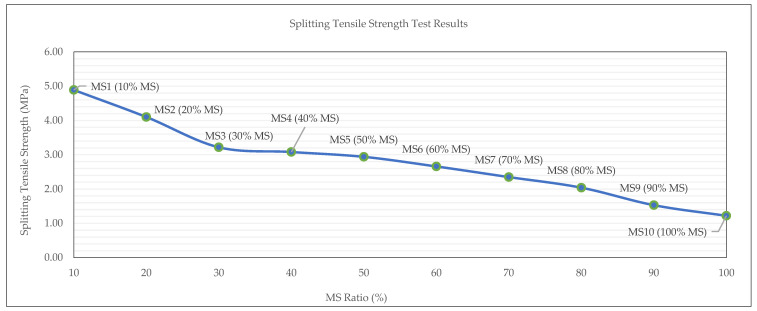
Splitting tensile strength test results of MS-based OPGC.

The results illustrated in Figure 11 show a clear inverse relationship between the MS content and the 28-day splitting tensile strength of OPGC mixtures. As the MS replacement ratio increased from 10% to 100%, the splitting tensile strength consistently decreased, dropping from 4.89 MPa at MS10 to 1.22 MPa at MS100. This decline parallels the trend observed in compressive strength, indicating that a higher MS content not only weakens the matrix in compression, but also compromises its tensile integrity. The reduction in tensile strength is attributed to the limited reactivity and poor gel formation ability of MS compared to more reactive binders like GGBS. With increasing MS content, the geopolymer matrix likely contains more internal voids and exhibits weaker inter-particle bonding, leading to a brittle failure under tensile stress. The steep decline after 50% MS further supports the conclusion that excessive MS content disrupts cohesive strength development within the binder phase.

Overall, these results suggest that high MS contents significantly reduce the tensile performance of OPGC, and that the material becomes unsuitable for structural applications requiring adequate tensile resistance. It reinforces the recommendation that MS should be used at replacement levels not exceeding 30% to maintain acceptable mechanical properties, as clarified in previous studies [56].

### 3.7. Flexural Strength

**Figure 12:**
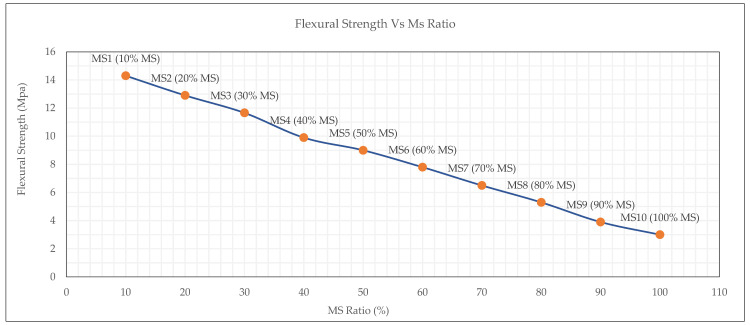
Flexural strength test results of MS-based OPGC.

The experimental results presented in Figure 12 illustrate the influence of MS content on the 28-day flexural strength of OPGC. A consistent downward trend was observed in the mechanical properties as the MS replacement ratio increased from 10% to 100%.

**Figure 13:**
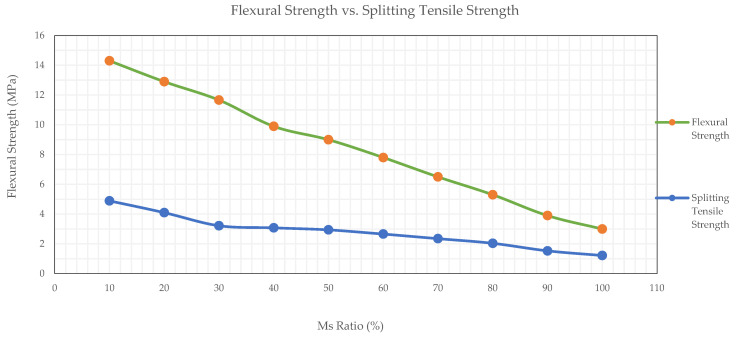
Comparison of 28-day flexural and splitting tensile strengths of MS-based OPGC.

Mixtures containing lower MS ratios (10–30%) exhibited superior flexural performance, reaching a maximum of 14.30 MPa at 10% MS, followed by 12.90 MPa and 11.66 MPa for 20% and 30% MS, respectively. Beyond this threshold, the flexural strength progressively declined. This behavior is attributed to the lower reactivity and gel-forming capacity of MS compared to GGBS, which limits the development of a dense and cohesive microstructure essential for flexural load resistance. The comparison graph in Figure 13 clearly highlights that flexural strength consistently exceeds splitting tensile strength across all mix compositions, with the gap widening at lower MS contents. This suggests that while both properties degrade with increasing MS, flexural behavior remains more resilient, due to its dependence on both matrix quality and aggregate bridging mechanisms [57]. These findings confirm that excessive MS replacement adversely impacts the tensile-related mechanical properties of OPGC. To maintain structural performance within acceptable limits, the MS content should be limited to 30% or below, especially in applications where flexural or tensile stresses are critical.

### 3.8. Scanning Electron Microscopy (SEM)

**Figure 14:**
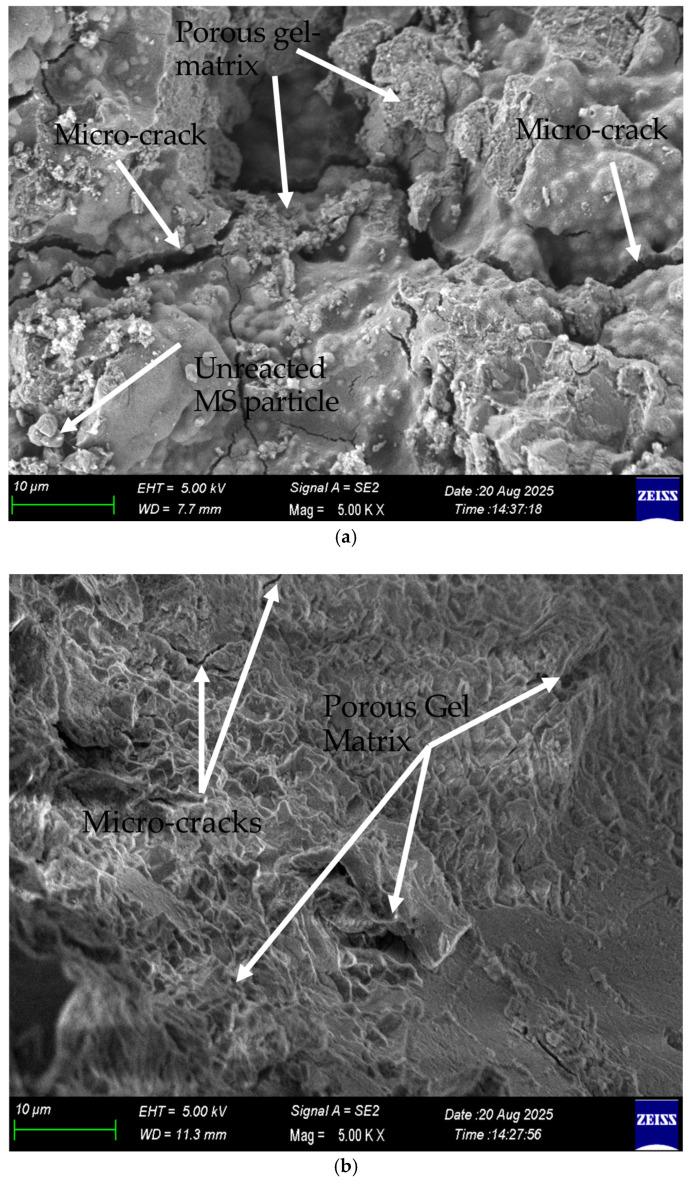

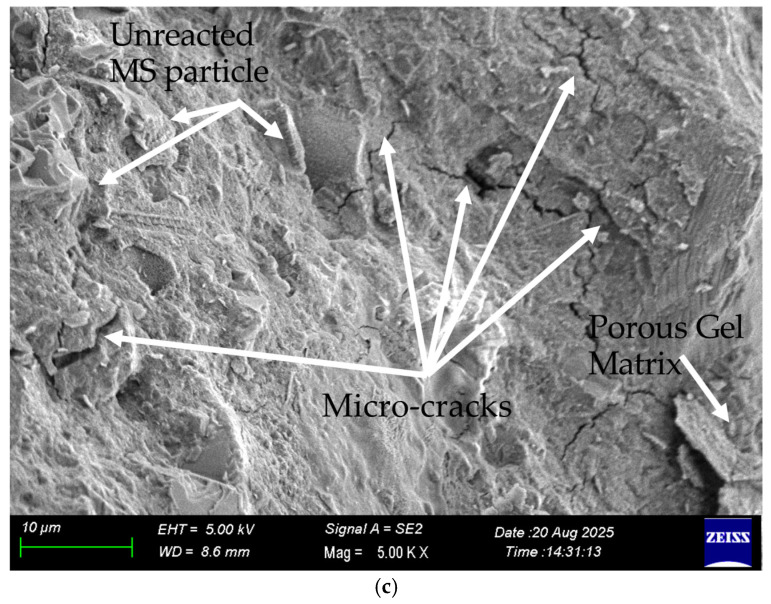
SEM micrograph of (**a**) 30% MS-based OPGC (**b**) 40% MS-based OPGC, (**c**) 50% MS-based OPGC.

Three selected OPGCs containing 30%, 40%, and 50% MS and solid activators, cured at 60 °C for 24 h, were examined using SEM to investigate their morphology and microstructure. The results of this analysis are presented in Figure 14. The SEM images were analyzed to gain a clearer understanding of the microstructural characteristics and morphology of the formed geopolymer. As shown in Figure 14a, the MS30 specimen exhibited a dense and compact matrix with very limited unreacted particles and microcracks. The homogeneously distributed reaction products indicate a well-developed geopolymer network. This microstructure suggests that the high calcium content of GGBS contributed to the formation of C–A–S–H and N–A–S–H gels, which are known to enhance matrix cohesion and strength [58]. Similar compact morphologies have been linked in the literature to higher compressive strength and lower porosity [28,59].

The MS40 specimen displayed a partially compact, yet fibrous and layered, microstructure, as shown in Figure 14b. The presence of these fibrous gel formations may indicate a slower dissolution of aluminosilicate species due to the increased MS content, leading to heterogeneity in the reaction products. Previous studies [46,60] have also reported that higher proportions of MS can decrease reactivity, resulting in localized microcrack formation and less uniform gel distribution. The coexistence of dense and porous regions observed here supports this explanation.

In the MS50 sample, microcracks and porous regions became more pronounced, as shown in Figure 14c. Several unreacted MS particles were clearly visible, suggesting incomplete geopolymerization. The reduction in calcium availability and the slower dissolution rate of MS likely limited the formation of binding gels, producing a weaker and more discontinuous matrix. These microstructural features correspond well with the observed reduction in compressive strength, consistent with earlier findings in [21,59].

The microstructural evolution observed in SEM images explains the strength reduction as the MS content increases from 30% to 50%. At 50% MS, a significant ‘pore structure change’ is evident, characterized by an increased volume of microvoids and visible microcracks compared to the 30% MS samples. This is attributed to the lower reactivity of MS relative to GGBS, leading to a ‘gel-phase transition’ where the binder matrix becomes less coherent and more fragmented.

Overall, as the MS content increased, the reactive Si and Al species available for geopolymerization decreased, leading to reduced gel formation and a less cohesive structure. Mixtures with up to 30% MS exhibited a dense, homogeneous matrix, while those with 50% MS displayed a heterogeneous and crack-prone morphology. This trend aligns with previous reports suggesting that maintaining MS content below 30% ensures adequate reactivity and mechanical performance [21,56].

## 4. Conclusions

The primary contribution of this research is the successful development of a sustainable, ‘just-add-water’ geopolymer using MS, providing a precise roadmap for its dosage limits in structural applications. By identifying the underlying relationship between MS mineralogy and geopolymerization kinetics, this study bridges the gap between industrial waste valorization and high-performance material design. Through a systematic investigation from the fresh state to the microstructural level, this research establishes a vital balance between sustainability and structural performance. Based on the comprehensive experimental findings, the following conclusions are drawn:

- Optimal substitution threshold: the study identifies a critical performance threshold at 30% MS replacement. Mixtures containing up to 30% MS achieved competitive mechanical properties, with compressive strengths exceeding 59 MPa at 28 days, comparable to high-performance conventional concrete. Beyond this limit, a significant degradation in the geopolymer matrix was observed due to reduced aluminosilicate reactivity.
- Fresh state behavior and workability: increasing MS content positively influences the workability of OPGC, with slump values rising from 7 cm at MS1 (10% MS) to 14 cm at MS10 (100% MS). This trend is directly linked to the lower inherent cementitious activity and specific mineralogical structure of MS (Q^0^ and Q^4^ units), which reduces the immediate water demand compared to highly reactive GGBS-rich systems.
- Setting time regulation: MS acts as an effective setting retarder in OPGC systems. The final setting time was extended by 320% when GGBS was fully replaced by MS. This finding is crucial for practical engineering applications, as it demonstrates that MS can be strategically used to control the rapid setting characteristic typically associated with alkali-activated GGBS concretes.
- Mechanical performance and matrix integrity: a strong correlation was established between density, ultrasonic pulse velocity (UPV), and mechanical strength. Although a steady decline in density and UPV values was observed starting from 10% MS, the impact of this reduction became critical beyond the 30% threshold. The inclusion of MS beyond this limit led to a more pronounced loss of matrix cohesion and increased porosity, as evidenced by the sharper drop in UPV. Splitting tensile and flexural strengths followed a similar downward trend, confirming that excessive MS substitution compromises the tensile integrity of the binder, leading to a less dense and more brittle internal structure.
- Microstructural validation: SEM analysis provided visual evidence of the 30% threshold revealing a dense and compact microstructure at MS3 (30% MS), whereas higher replacement levels exhibited microcracks and unreacted particles. While the MS30 specimen exhibited a dense, homogeneous C–(A)–S–H/N–(A)–S–H gel matrix, the MS50 sample showed pronounced micro crack-sand unreacted particles. This validates that sufficient calcium and reactive silica levels are maintained only at lower MS substitution ratios, ensuring a robust geopolymer network.

Despite the promising results, future research should focus on the long-term durability properties of MS-based one-part geopolymers, particularly their resistance to carbonation and chloride penetration. Additionally, investigating the life-cycle assessment (LCA) and the environmental impact of large-scale MS applications, as well as the potential of using chemical accelerators to enhance reactivity, will be essential to further validate the industrial feasibility and structural reliability of this sustainable binder.

## Figures and Tables

**Table 1:** Chemical compositions of MS and GGBS.

Component Name	MS (wt%)	GGBS (wt%)
SiO_2_	29.68	29.40
Al_2_O_3_	1.91	15.28
Fe_2_O_3_	4.29	1.30
CaO	51.70	42.10
MgO	9.93	7.01
SO_3_	0.52	2.87
Na_2_O	0.31	0.49
K_2_O	1.30	0.79
Cl	0	0.05
Loi	0.36	0.69

**Table 2:** The physical and chemical properties of the solid activator sodium metasilicate.

Properties	Value
Molecular structure	Composed of sodium (Na), silicon (Si), and oxygen (O) atoms
Components	Sodium oxide (Na_2_O): provides alkaline structure Silica (SiO_2_): promotes the formation of reactive phases
Physical form	White granules
Density (solid)	2.5 g/cm^3^
Solubility	Easily dissolves in water; dissociates into Na^+^ and SiO_3_^2−^ ions
Reactivity	Initiates the alkaline activation process Supports the formation of C-S-H and N-A-S-H gel phases in geopolymer concrete production
Moisture absorption	Rapidly absorbs moisture from the air; should be stored in a dry environment
Thermal stability	Stable up to 1088 °C

**Table 3:** The physical properties of the aggregates used in the experiment.

Properties	Value
Dry-specific gravity	2.56
Saturated surface dry-specific gravity	2.66
Water absorption	1.1

**Table 4:** The quantities of materials used in the OPGC experiment.

Materials’ Name	Quantities (kg/m^3^)
Coarse aggregate	961.0
Fine aggregate	693.0
Alumina–silicate	398.8
Binder	462.6

**Table 5:** The mixture design of MS-based OPGC.

	Material ID									
Materials (kg/m^3^)	MS1	MS2	MS3	MS4	MS5	MS6	MS7	MS8	MS9	MS10
Coarse aggregate	961.0	961.0	961.0	961.0	961.0	961.0	961.0	961.0	961.0	961.0
Fine aggregate	693.0	693.0	693.0	693.0	693.0	693.0	693.0	693.0	693.0	693.0
MS	39.9	79.8	119.6	159.5	199.4	239.3	279.2	319.0	358.9	398.8
GGBS	358.9	319.0	279.2	239.3	199.4	159.5	119.6	79.8	39.9	0.0
Activator	63.8	63.8	63.8	63.8	63.8	63.8	63.8	63.8	63.8	63.8
Water	217.4	217.4	217.4	217.4	217.4	217.4	217.4	217.4	217.4	217.4
Binder	462.6	462.6	462.6	462.6	462.6	462.6	462.6	462.6	462.6	462.6

**Table 6:** The slump results of MS-based OPGC.

Material ID	Slump Value (cm)	MS Value (%)	GGBS Value (%)
MS1	7	10	90
MS2	8	20	80
MS3	8	30	70
MS4	9	40	60
MS5	10	50	50
MS6	10	60	40
MS7	11	70	30
MS8	12	80	20
MS9	12	90	10
MS10	14	100	0

## Data Availability

The original contributions presented in this study are included in the article. Further inquiries can be directed to the corresponding author.

## Abbreviations

The following abbreviations are used in this manuscript:

MS	Magnesium slag
OPGC	One-part geopolymer concrete
GGBS	Ground granulated blast furnace slag
UPV	Ultrasonic pulse velocity
